# Successful closed reduction for iatrogenic displacement of the anatomical neck of the humerus: a case report

**DOI:** 10.1186/1756-0500-7-770

**Published:** 2014-11-03

**Authors:** Masao Koda, Junshiro Hisamitsu, Shiro Nakayama, Satoru Nishikawa, Takeo Furuya, Masashi Yamazaki, Shuhei Ogino

**Affiliations:** Department of Orthopaedic Surgery, Prefectural Togane Hospital, 1229 Daikata, Togane, Chiba 2830811 Japan; Department of Orthopaedic Surgery, Chiba University Graduate School of Medicine, 1-8-1 Inohana, Chuo-Ku, Chiba 2608670 Japan; Department of Orthopaedic Surgery, Nishikawa Ortopaedic Clinic, 1-14-2 Osakidai, Sakura, 2850817 Japan; Department of Orthopaedic Surgery, University of Tsukuba, 1-1-1 Tennodai, Tsukuba, 30585775 Japan

**Keywords:** Iatrogenic displacement, Humeral anatomical neck, Fracture-dislocation of the shoulder

## Abstract

**Background:**

We report a rare case in which closed reduction was successfully obtained for iatrogenically displaced fracture-dislocation of the humeral anatomical neck with a favorable clinical outcome.

**Case presentation:**

A 53-year old postman suffered from shoulder dislocation with an undisplaced fracture of the humeral anatomical neck which was initially undiagnosed. After the first attempt to reduce the dislocation of the shoulder joint by Stimson’s method, complete displacement of the fractured humeral anatomical neck occurred. By closed reduction under general anesthesia, the displaced humeral head was successfully reduced and was subsequently treated by conservative therapy using sling immobilization. Follow-up by MRI two years later showed no evidence of avascular necrosis of the humeral head. The patient showed a satisfactory range of motion of the affected shoulder joint.

In the present case, the blood supply was partially preserved because a part of the lesser tubercle remained attached to the displaced humeral head.

**Conclusion:**

Based on this experience, we concluded that closed reduction might be attempted before deciding to perform an open reduction and internal fixation for displaced fracture-dislocation of the humeral anatomical neck.

## Background

Dislocation of the shoulder is commonly treated by closed reduction with or without sedation. In cases in which there is concomitant proximal humeral fracture with dislocation of the shoulder, fluoroscopy-assisted closed reduction under general anesthesia is recommended [1]. However, fracture of the humeral anatomical neck is occasionally disregarded because it is difficult to detect using only plain radiographs [2, 3].

A neglected fracture of the humeral anatomical neck might cause iatrogenic displacement of the humeral head during the reduction procedure. It is widely known that fracture of the humeral anatomical neck often results in avascular necrosis of the humeral head as a sequel [4, 5]. Moreover, a displaced fracture of the humeral anatomical neck often develops avascular necrosis even after successful open reduction and internal fixation, resulting in a poor clinical outcome. A report by Hersche et al showed that avascular necrosis of head of the humerus occurred after reduction in all six patients with iatrogenic displacement of a fracture-dislocation of the humeral anatomical neck [3]. Thus treatment of the displaced fracture-dislocation of the humeral anatomical neck remains an intractable problem. Here we report the case of iatrogenic displacement of a fracture-dislocation of the humeral anatomical neck that could be successfully reduced by the closed method, resulting in a favorable result.

## Case presentation

**Figure 1:**
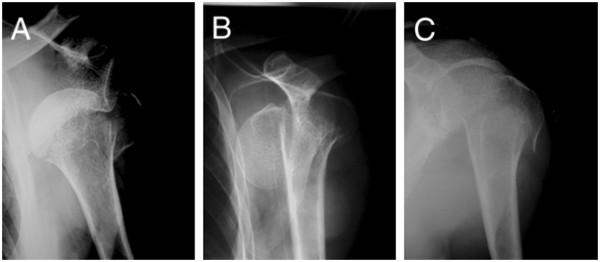
**X-ray findings.** Initial X-ray showed dislocation of the left shoulder, displaced fracture of the greater tuberosity and an undisplaced humeral anatomical neck fracture **(A)**. After an initial trial for reduction by Stimson’s method, X-ray showed the complete displacement of the humeral head **(B)**. After the fluoroscope-assisted closed reduction under general anesthesia, reduction was obtained with angulation between the humeral head and shaft **(C)**.

A 53-year old postman was injured in a bicycle accident. On admission, he complained of left shoulder pain and was unable to elevate his left arm. Initial X-ray showed a dislocation of the left shoulder and displaced fracture of the greater tuberosity (Figure 1A). There was an undisplaced humeral anatomical neck fracture that was initially undiagnosed and was retrospectively detected. First, we tried a closed reduction with Stimson’s method under pentazosine analgesia. After an evident click, his pain was reduced. A second X-ray was obtained to confirm the reduction. The second X-ray showed complete displacement of the humeral head (Figure 1B). The X-ray revealed that Neer’s classification was not applicable for the present patient’s fracture, but the present fracture was classified to C3 in AO classification.

Then the patient underwent fluoroscope-assisted closed reduction under general anesthesia. First, we manually pushed the displaced humeral head upward from the axilla to prevent further displacement. Next, we elevated the left arm laterally with traction to avoid a downward migration of the humeral head. When abduction reached approximately 90 degrees, we pushed the humeral shaft into contact with the humeral head with the manual push of the humeral head from axilla, resulting in a reduction of both displaced fragments (Figure 1C). Fluoroscopic examination immediately after the reduction revealed the contact between the humeral head and shaft. There was no instability with shoulder motion up to 60 degrees of abduction/flexion and 20 to 30 degrees of internal/external rotation.

**Figure 2:**
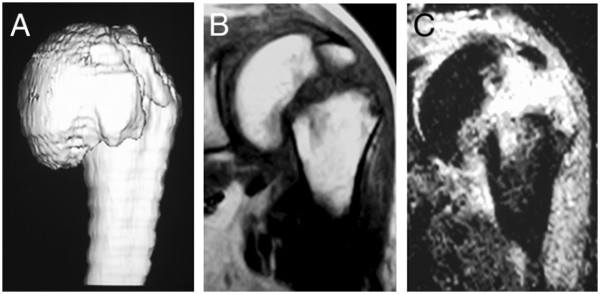
**3D-CT and MRI findings 4 weeks after the injury.** 3D-computed tomography showed an angulation deformity between humeral shaft and head of about 45° **(A)** and MRI showed no evidence of avascular necrosis of the humeral head **(B, C)**.

After closed reduction, the left arm was immobilized in a sling for four weeks, followed by pendulum exercise, assistive range-of-motion (ROM) exercise and active/passive ROM exercises. Four weeks after injury, MRI showed no evidence of avascular necrosis and 3D-computed tomography showed an angulation deformity between the humeral shaft and head of about 45° (Figure 2A). The clinical course was uneventful. At the final follow-up (two years after injury), the patient had no complaint except for slight limitation of lateral elevation of the left arm (anterior elevation: 150°, lateral elevation: 125°, internal rotation: T12 level) revealed by physical examination, and he completely returned to his previous work. X-ray showed complete union of the fragments with an angulation deformity and MRI showed no evidence for avascular necrosis of the humeral head (Figure 2B, C).

## Conclusions

Displaced humeral anatomical neck fracture is generally treated by open reduction and internal fixation [2]. However, open reduction and internal fixation can disrupt surrounding soft tissues attached to the humeral head, resulting in avascular necrosis of the humeral head. Avoiding open reduction might result in preservation of blood flow to the humeral head. Gerber reported a rare case in which fracture-dislocation of the humeral anatomical neck was treated by open reduction and internal fixation without consequent avascular necrosis of the humeral head [6]. In the present case, the blood supply was partially preserved because a part of the lesser tubercle remained attached to the displaced humeral head [7].

Based on this experience, we concluded that closed reduction might be attempted before deciding to perform an open reduction and internal fixation for displaced fracture-dislocation of the humeral anatomical neck.

As for methods of reduction of shoulder dislocation, iatrogenic displacement of the anatomical neck of the humerus was induced by Kocher’s maneuver in the previous reports and Stimson’s maneuver in the present case. It is still unclear which maneuver of reduction for shoulder dislocation is most suitable, iatrogenic displacement can occur by insufficient muscle relaxant and rough maneuver without general anesthesia.

## Consent

Written informed consent was obtained from the patient for publication of this Case report and any accompanying images. A copy of the written consent is available for review by the Editor of this journal.
